# Risk Assessment of Global Animal Melioidosis Under Current and Future Climate Scenarios

**DOI:** 10.3390/ani15030455

**Published:** 2025-02-06

**Authors:** Suya Li, Le Xu, Yuqing Jiao, Shiyuan Li, Yingxue Yang, Feng Lan, Si Chen, Churiga Man, Li Du, Qiaoling Chen, Fengyang Wang, Hongyan Gao

**Affiliations:** Hainan Key Laboratory for Tropical Animal Breeding and Disease Research, School of Tropical Agriculture and Forestry, Hainan University, Haikou 570228, China; 22210905000005@hainanu.edu.cn (S.L.); 22220952000023@hainanu.edu.cn (L.X.); 23210905000003@hainanu.edu.cn (Y.J.); 22lishiyuan@hainanu.edu.cn (S.L.); 23220952000023@hainanu.edu.cn (Y.Y.); 23220952000014@hainanu.edu.cn (F.L.); chensi.ruth@hotmail.com (S.C.); manchuriga@163.com (C.M.); kych2008d@163.com (L.D.); chenqiaoling@hainanu.edu.cn (Q.C.)

**Keywords:** animal melioidosis, maximum entropy model, future climate, risk factors

## Abstract

Melioidosis is a zoonotic disease that is caused by *Burkholderia pseudomallei*, which is a serious public health and safety risk. In order to explore the global animal melioidosis risk distribution and its dynamic response to future climate scenarios, we collected global data about reported animal incidence sites. Data regarding the density of *Burkholderia pseudomallei* in the environment were created by collecting and sorting information about the *Burkholderia pseudomallei* occurrence sites in contaminated air, soil, and water. Combined with bioclimatic variables, the maximum entropy (MaxEnt) niche was modeled for global animal melioidosis. The findings indicate that under current bioclimatic conditions, global animal melioidosis risk regions are concentrated between 30° S and 30° N, with high-risk areas being mainly in Central America, the northern part of South America, and eastern and southern India, among others. Most countries will expand their risk regions under future climatic scenarios. Melioidosis risk expanding towards higher northern latitudes has led to new epidemic areas. In addition, the melioidosis risk area will contract in some areas. Therefore, we have provided a basis for global melioidosis surveillance and propose feasible strategies for prevention and control in high-risk regions, which will help countries to carry out targeted surveillance and prevention to reduce risks and losses.

## Simple Summary

Melioidosis is a zoonotic disease caused by *Burkholderia pseudomallei.* To explore the global animal melioidosis risk distribution and its dynamic response under future climate scenarios, we collected details about the occurrence sites of animal melioidosis and the *Burkholderia pseudomallei* occurrence sites in contaminated air, soil and water. Maximum entropy (MaxEnt) was used to establish the melioidosis risk model. The results show that under current bioclimatic conditions, animal melioidosis high-risk regions are concentrated between 30° S and 30° N, with particularly high levels of risk in Central America, the northern part of South America, and eastern and southern India, among others. With future climate change, the risk regions in most countries are expected to expand, and new epidemic zones will emerge at higher northern latitudes. However, some areas may experience a contraction of risk regions. This study provides a basis for global melioidosis surveillance and offers effective early warning measures, which can help reduce losses in risk areas.

## 1. Introduction

Melioidosis is a zoonotic disease that results from an infection caused by *Burkholderia pseudomallei* (*B. pseudomallei*). Melioidosis was first reported in sheep in Australia in 1949 [1], while the first human case occurred in 1950 [2]. Melioidosis commonly occurs in Southeast Asia and northern Australia [3,4], with sporadic cases in Africa, the USA, and the Middle East [5]. Limmathurotsakul et al. found that *B. pseudomallei* can infect a variety of animals, including goats, pigs, cows, horses, monkeys, and even fish, among which goats are the most severely infected [6]. Clinical signs of infection include lameness, hind leg paralysis, severe mastitis, pneumonia, and abortion in ewes [7]. Diseased animals are a potential source of zoonotic infection for humans through the excretion of *B. pseudomallei* in feces, which can contaminate air, drinking water, and soil. Reinfection may occur when healthy animals or humans are exposed to drinking water and aerosols [8] or when broken skin is exposed to contaminants [9,10]. Therefore, the infection rate of melioidosis has obvious spatial heterogeneity in different regions and is affected by various factors, such as regional environment, climate, health conditions of residents, and animal density [11]. Several studies have shown that climate factors such as rainfall, temperature, and humidity have a significant impact on the incidence rate of melioidosis [12]. In particular, extreme weather events caused by climate change, such as floods and typhoons, further increase the risk of the transmission of melioidosis [13] Therefore, understanding the link between melioidosis and climate change is crucial for predicting and preventing the spread of melioidosis.

The epidemiological patterns of *B. pseudomallei* must be established for the prevention and control of melioidosis. Many studies use modeling methods to support epidemiological studies with missing information. Ganeshalingam et al. [14] established a negative binomial regression model to assess the association between the incidence of melioidosis and various meteorological factors in human populations in the Townsville region of Australia and found that the local incidence of melioidosis increased several times due to prolonged rainfall. Kaestli et al. [15] used boosted regression tree and negative binomial modeling and found a significant increase in the number of cases of melioidosis in the population in the Darwin region of Australia when the daily maximum temperature increased, especially when this coincided with rainfall. Shaw et al. [16] found, through binomial logistic regression modeling, that *B. pseudomallei* was widely distributed and most prevalent in the soil during the rainy season in southwestern India. Thus, to some extent, precipitation and temperature influence the distribution and abundance of *B. pseudomallei*, and, accordingly, the prevalence and distribution of melioidosis. The Sixth Assessment Report of the Intergovernmental Panel on Climate Change reported that global warming will exceed 1.5 °C in the 21st century due to cumulative emissions of greenhouse gases [17]. Global warming is expected to cause a further increase in average terrestrial rainfall [18,19,20], raising concerns about the impact of climate change on the spread of melioidosis [5,21].

The introduction of more comprehensive models, such as ecological niche models (ENMs), has facilitated the evaluation of the potential environmental adaptability of a species using current data on species distribution and relevant environmental factors [22]. In recent years, the maximum entropy (MaxEnt) model has been extensively employed in predicting the risk of animal diseases [23,24,25,26]. Studies have shown that the MaxEnt model outperforms other ecological niche models in terms of prediction accuracy and stability [27]. Furthermore, it is preferred for its simplicity, speed, and minimal sample requirements, making it an ideal tool for many researchers [28]. The model can be run even with minimal occurrence data, and its performance can be evaluated [29].

Animals infected with *B. pseudomallei* are important links in the transmission of melioidosis. For example, the international movement of animals with melioidosis has been associated with cases in non-endemic regions. However, no global risk assessment of animal melioidosis has been conducted. To estimate the risk distribution of animal melioidosis and its response to future climate change using empirical methods, this study used the MaxEnt niche model to predict the global risk of animal melioidosis under current and future climate scenarios (2050s, 2070s, and 2090s). The findings offer insights into the geographical distribution and epidemic trend of animal melioidosis and provide a scientific basis for developing targeted prevention and control strategies.

## 2. Materials and Methods

### 2.1. Occurrence Sites of Animal Melioidosis

Data on animal melioidosis occurrence sites (1925–2024) were retrieved from databases such as China National Knowledge Infrastructure, Wanfang Database, and PubMed. Literature search was performed on the aforementioned search engines using keywords such as “Melioidosis”, “*Burkholderia pseudomallei*”, and “animals”. Keywords were searched using “AND” or “OR” logic. In cases where records lacked coordinates, Google Maps was used to acquire them. In total, 138 animal incidence sites were identified.

**Figure 1:**
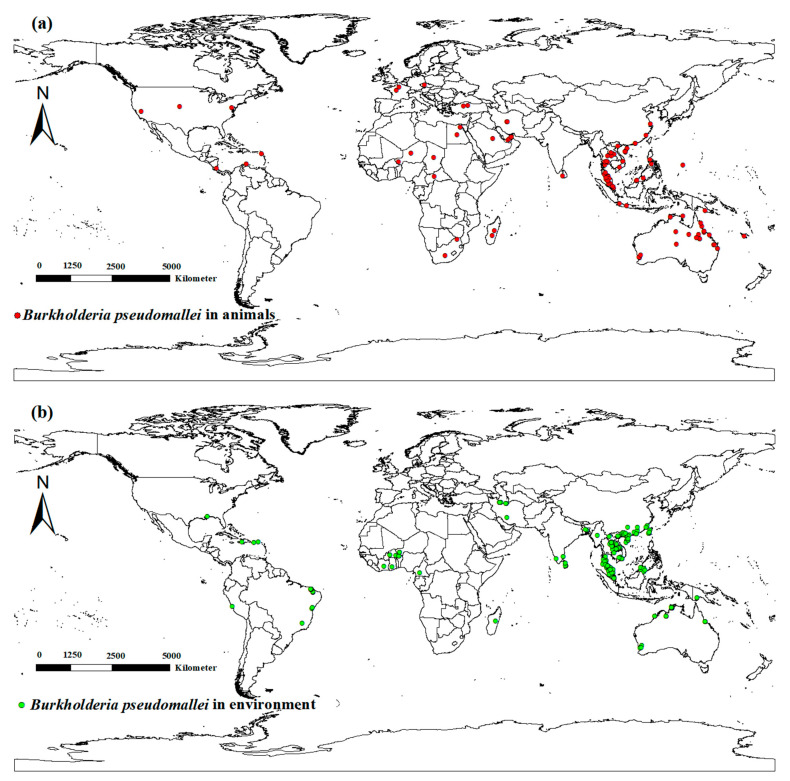
Global distribution of *Burkholderia pseudomallei*. (**a**) *B. pseudomallei* in animals and (**b**) *B. pseudomallei* in the environment. The world standard map was downloaded from the Resource and Environmental Science Data Center at the Chinese Academy of Sciences.

To mitigate spatial bias and eradicate spatial autocorrelation, we used SDM toolbox (v2.2; http://www.sdmtoolbox.org/, accessed on 3 December 2024) in the ArcGIS 10.2 toolbox to filter the animal incidence locations to within a 1 km radius. Additionally, imported cases were removed from the filtering process. A total of 100 animal melioidosis incidence sites were ultimately identified (Figure 1a).

### 2.2. Collection and Processing of Environmental Variables

Considering the impact of *B. pseudomallei* in the environment on the spread of melioidosis, we recorded details of *B. pseudomallei* occurrence sites in air, water, and soil from the published literature. Data on occurrence sites where *B. pseudomallei* existed in air, water, and soil were retrieved from databases such as China National Knowledge Infrastructure, Wanfang Database, and PubMed. These occurrence sites where *B. pseudomallei* existed in air, water, and soil were collectively classified as environmental occurrence sites. In total, 176 occurrence sites were identified (Figure 1b). After obtaining the location coordinates of *B. pseudomallei* occurrence sites, kernel density analysis was performed in ArcGIS 10.2 to obtain the environmental density of *B. pseudomallei*.

Bioclimatic variables are commonly used in ecological niche modeling. They provide a comprehensive summary of global temperature and precipitation conditions that indicate the meteorological factors that influence the ecological adaptability of diseases, pathogens, and species in modeling [30]. These variables are commonly used in ENMs of animal diseases [31,32,33]. We simulated current global climate conditions using 19 bioclimatic variables (bio 1–19) obtained from WorldClim (version2.1; https://worldclim.org/data/worldclim21.html#, accessed on 3 December 2024).

To simulate future climate scenarios, climate data for the 2050s (2041–2060), 2070s (2061–2080), and 2090s (2081–2100) were obtained from the Beijing Climate Center Climate System Model Medium Resolution dataset, which is a part of the Coupled Model Intercomparison Project Phase 6. This study used three shared socioeconomic pathways (SSPs), namely, sustainable development (SSP 126), intermediate development (SSP 245), and conventional development (SSP 585) [34], which were obtained from https://worldclim.org/data/cmip6/cmip6climate.html (accessed on 3 December 2024). The SSPs offer more comprehensive insights into the relationship between socioeconomic development and climate scenarios [35].

### 2.3. Eliminating Spatial Autocorrelation of Environmental Variables

Data on environmental variables are essential for constructing niche models. However, incorporating an excessive number of these variables may intensify spatial correlation among the variables, leading to overfitting [36]. Therefore, assessing multicollinearity between variables before model construction is essential. Spearman correlation analysis was performed on 19 bioclimatic variables using SPSS software (IBM SPSS Statistics V22.0). To reduce redundancy, in case of a strong correlation between two variables, the variable with the lower contribution rate to the model and Spearman coefficient was eliminated. Thus, only environmental variables with correlation coefficients <0.8 were included in the MaxEnt model [37]. The variables included in the model are shown in Table 1.

### 2.4. Establishment of the MaxEnt Model of Animal Melioidosis

The MaxEnt model is based on the concept of maximizing entropy to produce a probability distribution that aligns with the known occurrence locations, while ensuring a uniform probability distribution, subject to certain constraints. Here is the mathematical formulation of the MaxEnt model [38,39]:

(1) $$P_{\omega }\left(y|x\right)=\frac{1}{Z_{\omega }\left(x\right)}exp\left(\sum_{i=1}^{n}\omega ifi\left(x|y\right)\right)$$

(2) $$Z_{\omega }\left(x\right)=\sum_{y}exp\left(\sum_{i=1}^{n}\omega ifi\left(x|y\right)\right)$$

where *x* represents the environmental variable input into the model; *y* represents the predicted geographical area; $fi\left(x|y\right)$ represents the feature functions; ωi represents the weights associated with $\;fi\left(x|y\right)$; and $Z_{\omega }\left(x\right)$ indicates the normalization constant. 

In this study, the niche modeling software MaxEnt version 3.4.1 was used to establish the melioidosis risk model. The refined data on animal melioidosis incidence sites, along with selected current bioclimatic variables and the environmental density of *B. pseudomallei*, were inputted into the MaxEnt model to generate a global potential risk map of melioidosis under current climate conditions. To create a robust model, the program randomly allocated 25% of the melioidosis incidence sites for testing, while the remaining 75% served as the training set. To mitigate sampling bias, 10,000 background points were randomly selected as pseudo-absence data. The modeling process was repeated 10 times, using the subsample selection method for repeated runs. Finally, the average values were used as the basis for further analysis, resulting in the construction of the final MaxEnt model used in this study.

To simulate future climate scenarios, the MaxEnt model was run using data from SSP 126, 245, and 585 to generate a global potential risk map for melioidosis under various future climate scenarios.

The global melioidosis risk areas were marked on the map using ArcGIS 10.2. The standard world map was downloaded from Resource and Environmental Science Data Center at the Chinese Academy of Sciences (http://www.resdc.cn/, accessed on 3 December 2024). The figure number of the map is GS[2016]1666, and it was not modified.

### 2.5. Model Evaluation and Interpretation

The jackknife test evaluates the training gain of a model when each variable is applied individually. In recent years, receiver operating characteristic (ROC) curve analysis has been widely used for evaluating potential species distribution prediction models [40]. The ROC curve of experimental data is used to evaluate the effectiveness of the model, whereas the area under the curve (AUC) is used to evaluate prediction accuracy. Because AUC values are not influenced by thresholds, the assessment is more objective [41,42]. The AUC value ranges from 0 to 1. Generally, in maximum entropy modeling, the closer the AUC is to 1, the more reliable the model is [43]. When the AUC value is 0.7–0.8, the model simulation performance is acceptable. An AUC value of 0.8–0.9 indicates excellent model simulation performance, whereas an AUC value >0.9 indicates outstanding simulation performance. The global risk of animal melioidosis is represented by a scale of 0–1. ArcGIS 10.2 was utilized to visualize the global potential risk map of melioidosis.

## 3. Results

### 3.1. Environmental Variables Used in the Animal Melioidosis Model

**Figure 2:**
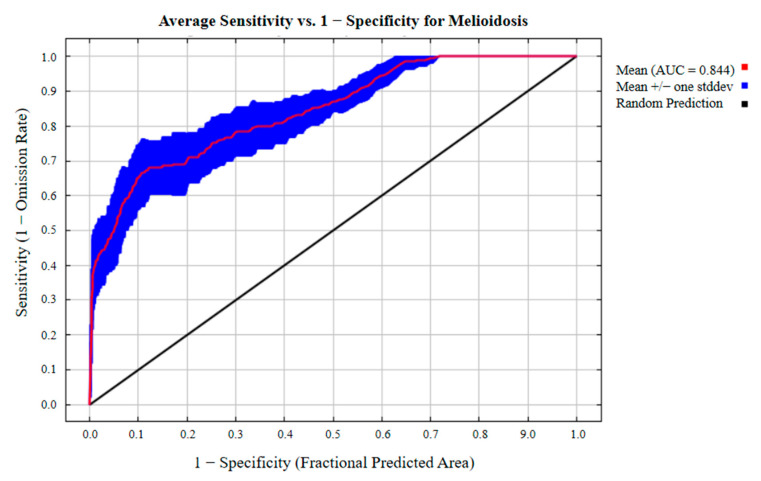
Receiver operating characteristic curve of the animal melioidosis model.

The global animal melioidosis model constructed under current climatic conditions exhibited high accuracy. This model had a mean ± standard deviation AUC value of 0.844 ± 0.034 (Figure 2). This result demonstrated strong confidence in the capability of the MaxEnt model to forecast the worldwide spread of animal melioidosis.

**Figure 3:**
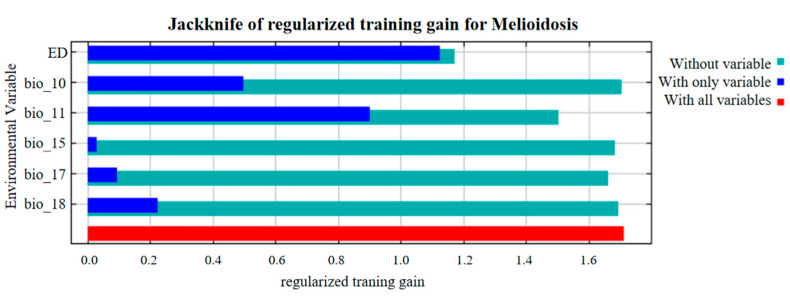
Jackknife test results of the animal melioidosis model.

In the jackknife test, the vertical axis indicates the selected environmental variables, while the horizontal axis indicates the regularized traning gain for each environmental variable (Figure 3). The results of the jackknife test revealed that the density of *B. pseudomallei* in the environment provided the highest benefit among the environmental variables, indicating its significant impact on the potential global distribution risk of animal melioidosis. This is followed by the mean temperature of the coldest quarter (bio 11), the mean temperature of the warmest quarter (bio 10), precipitation in the warmest quarter (bio 18), precipitation in the driest quarter (bio 17), and precipitation seasonality (bio 15). The sum contribution of the four most important variables is 98.7%.

**Figure 4:**
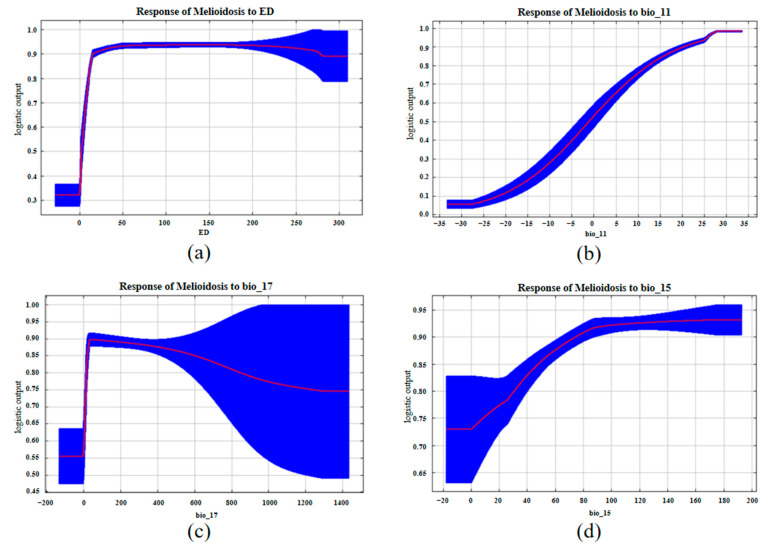
Response curves of important variables in the animal melioidosis model. (**a**) Environmental density of *Burkholderia pseudomallei*; (**b**) mean temperature of the coldest quarter; (**c**) precipitation in the driest quarter; and (**d**) precipitation seasonality. Red is the response curve, blue is the standard error.

Figure 4 depicts the response curves for the effects of important environmental variables on the probability of melioidosis occurrence. According to the response curve of the environmental density of *B. pseudomallei*, the density index range was 0–18. The risk of melioidosis occurrence gradually increased with the environmental density of *B. pseudomallei* (Figure 4a). The response curve of the mean temperature of the coldest quarter indicated a positive correlation with the risk of melioidosis occurrence, which increased with the mean temperature (Figure 4b). The response curve of the precipitation in the driest quarter showed that the risk of melioidosis occurrence increased rapidly when the precipitation was 0–45 mm and then showed a slow downward trend (Figure 4c). A positive correlation was found between precipitation seasonality and the overall risk of melioidosis occurrence, which increased gradually with the precipitation seasonality (Figure 4d).

### 3.2. Global Risk Regions for Animal Melioidosis

**Figure 5:**
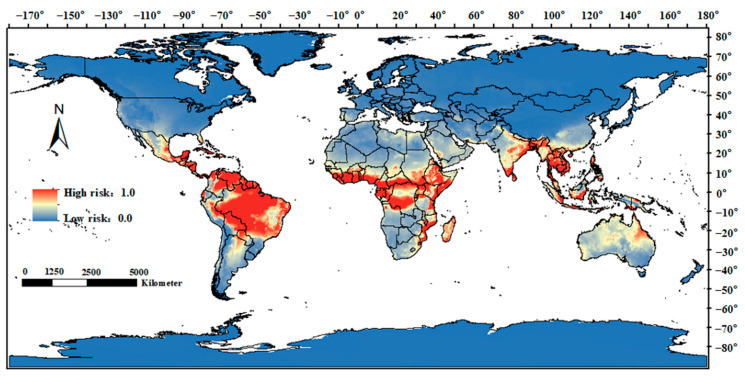
Global risk regions for animal melioidosis under current climate conditions. The red areas represent high-risk areas, where the probability of *B. pseudomallei* infection is highest, and the blue areas represent low-risk areas. The world standard map was downloaded from the Resource and Environmental Science Data Center at the Chinese Academy of Sciences.

As a result of current climate conditions, the global animal melioidosis risk regions are concentrated between 30° S and 30° N, with high-risk regions being distributed in Central America, the northern region of South America, the central and eastern coastal regions of Africa, southern and eastern India, almost all of Southeast Asia, the southern coastal regions of China, and Queensland, Australia. (Figure 5).

### 3.3. Risk Distribution Changes for Animal Melioidosis Under Future Climate Scenarios

**Figure 6:**
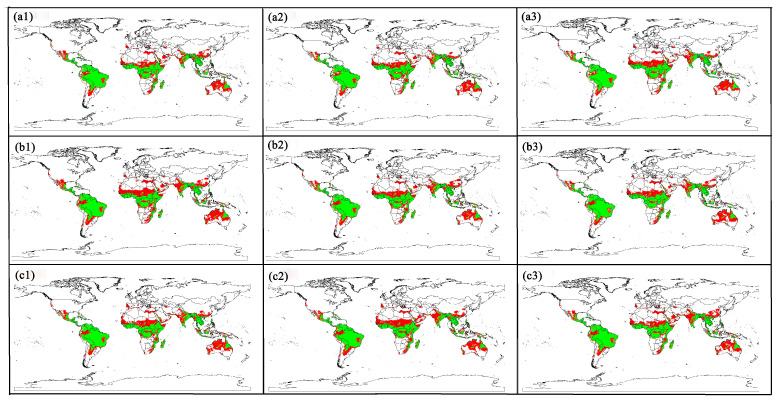
Distribution changes between current and future climatic scenarios for melioidosis. (**a1**): 2050s-SSP 126; (**a2**): 2070s-SSP 126; (**a3**): 2090s-SSP 126; (**b1**): 2050s-SSP 245; (**b2**): 2070s-SSP 245; (**b3**): 2090s-SSP 245; (**c1**): 2050s-SSP 585; (**c2**): 2070s-SSP 585; and (**c3**): 2090s-SSP 585. Red indicates the regions of future expansion; green indicates the regions of current climatic scenarios; and blue indicates the regions of future contraction. The world standard map was downloaded from the Resource and Environmental Science Data Center at the Chinese Academy of Sciences.

With future climate change, the potential risk regions for animal melioidosis will increase to varying degrees, with a trend toward expanding to high latitude regions (Figure 6). Compared with low-risk regions under the current climate conditions, risk regions in most countries will continuously expand. These regions include Sonora, Coahuila, Nuevo León, Guadalajara, Colima, Aguascalientes, Apachingan, and Hidalgo in Mexico; Miriti Parana and La Pedrera in Colombia; Northern Loreto and the Northern Amazon Provinces in Peru; Tocantins and Goiás in Brazil; Corrientes, Santa Fe, Cordoba, and Provincia de Salta in Argentina; Oasis Province in Libya; Cairo in Egypt; Nouakchott, Rischetole, and Butilimit in Mauritania; Senegal; Bamako in La République du Mali; Niamey, Agadez, Région de Tahoua, Maradi, Zinder, Région de Diffa, Région de Dosso, and Gaya in Niger; Sokoto, Zamfara, Kastina, Kanon, Jigawa, Yobe, and Borno States in Nigeria; Kanem, Batha, Biltine, Ouaddai, Salamat, and Logone Oriental and Occidental in Chad; Northern Darfur, Kordofan, Sennar, and Blue Nile in Sudan; Hargeisa, Ceerigaabo, and Garowe in Somalia; Basoko and Kisangani in Congo; Republic of Rwanda; Maseru, Fixburg, and Botshabelo in South Africa; Toliara in Madagascar; Jaipur, Jodhpur, Udaipur, Aurangabad, Group of Monuments at Hampi, and Khajrāho in India; Magway and Mandalay in Myanmar; Sichuan, Guangdong, Guizhou, and Yunnan Provinces in China; Uluru-Kata Tjuta National Park, Alice Springs, Queensland, and Brisbane in Australia; and Northern and Western Australia.

Notably, as the melioidosis risk regions have expanded toward northern latitudes, many new epidemic regions have emerged, including Salem in the USA, Portugal, Extremadura in the Kingdom of Spain, Sardinia in Italy, and the Caspian Sea. In addition, melioidosis risk will occur in areas such as Iran, Brunei, and western Niger.

Under SSP 126, the risk expansion regions of global animal melioidosis in the 2050s, 2070s, and 2090s were comparable to those of global animal melioidosis under current climate conditions. The risk expansion regions of global animal melioidosis in the 2050s under SSP 245 were larger than those in the 2070s and 2090s, indicating that global animal melioidosis risk is higher in the 2050s under SSP 245. The risk expansion regions of global animal melioidosis were the largest in the 2070s under SSP 585. The frequency and severity of melioidosis could increase in the 2070s under SSP 585. In addition, for the 2070s, the expansion regions of global animal melioidosis under SSP 585 were larger than those under SSP 126 and SSP 245, which can be explained by the larger degree of climate change under SSP 585 than under SSP 126 and SSP 245 (Table 2). 

## 4. Discussion

Melioidosis is a zoonotic disease that cannot be ignored, as both animals and humans are generally susceptible to *B. pseudomallei* infection. Although direct zoonotic transmission (animal-to-person) and person-to-person transmission of melioidosis is regarded as extremely rare, under certain conditions, such as after a strong typhoon, the density of *B. pseudomallei* in the environment will significantly increase. This creates suitable conditions for the zoonotic transmission of *B. pseudomallei*, increasing the probability and intensity of a potential epidemic. The introduction of *B. pseudomallei* into nonendemic countries will jeopardize human health and the development of animal husbandry industries. In 1975, several zoos in France were struck by melioidosis outbreaks, resulting in the death and slaughtering of several animals as well as the death of two humans [44]. In 1992, an outbreak of melioidosis was reported in primates imported from the Philippines to the United Kingdom [45]. In recent years, many cases of imported animals developing melioidosis after a long latency period have been reported [46,47]. Melioidosis is an informally neglected tropical disease in the scientific community [48]. Due to the gradual increase in global melioidosis cases, the distribution of *B. pseudomallei* through zoonotic transmission merits attention. Therefore, the potential risk of global animal melioidosis needs to be accurately anticipated.

Using the MaxEnt niche model, we constructed a global animal melioidosis risk map under current climate conditions. This map estimated that the potential risk regions of animal melioidosis were mainly distributed in the 30° N–30° S region. In fact, *B. pseudomallei* is widely distributed in tropical regions between 20° N and 20° S [49]. The majority of the region’s poor inhabitants are engaged in agricultural work, which involves raising a large number of animals, and lack awareness regarding the risk of melioidosis [50,51]. Therefore, the proportion of human and animal infections in these regions is relatively high [48]. The potential risk regions highly overlap with the distribution range of *B. pseudomallei*. Meanwhile, the response curves showed that animal melioidosis is more likely to occur in a warm climate with abundant precipitation and seasonal variations, which is precisely that found in the geographical region of 30° N–30° S. These data demonstrate the good applicability of the animal melioidosis model constructed in this study.

The environmental density of *B. pseudomallei* is the major risk factor for animal melioidosis because *B. pseudomallei* is the pathogen that causes melioidosis. Limmathurotsakul et al. predicted that the probability of goats being infected with melioidosis is similar to that of humans, possibly because goats and humans have similar opportunities to be exposed to *B. pseudomallei* in similar environments [6]. In addition, it is important to note that precipitation affects the distribution of *B. pseudomallei*. For example, in areas where the disease is endemic, such as Thailand and Singapore, rainfall is a triggering factor [52]. Chen et al. [53] showed that rainfall was positively correlated with the presence of the *B. pseudomallei* in the environment in Kaohsiung. In northern Australia, the incidence rate of melioidosis increases by 14% for every 100 mm of rainfall [54]. In addition, a study showed that the high humidity in Laos and Cambodia facilitated the formation of inhalable bacterial aerosols, which, significantly, led to seasonal outbreaks of melioidosis [55]. Therefore, higher precipitation will create humid environmental conditions, which facilitate the spread of *B. pseudomallei*. Furthermore, *B. pseudomallei* can be isolated from moist, clay-rich soil and accumulated surface water, and its proliferation thrives with a high soil moisture content [56]. When the soil moisture content is below 10%, *B. pseudomallei* die within 70 days. Conversely, when the moisture content exceeds 40%, *B. pseudomallei* can remain viable for 726 days [57]. Therefore, after heavy rain, people and animals should avoid contact with contaminated soil and water sources and take measures to prevent infection through *B. pseudomallei* aerosols.

However, temperature affects the living conditions and distribution changes of *B. pseudomallei*. When the temperature in the environment is 0 °C, *B. pseudomallei* dies within 18 days, whereas at 24–32 °C, *B. pseudomallei* grows vigorously [57]. On a large spatial scale, the sustained rise in global temperature will lead to increased rainfall in high latitude regions, and climate change will induce the migration of animal species to higher altitudes or latitudes [58,59,60]. The predicted melioidosis risk under future climate scenarios shows that with global warming, the suitable area for *B. pseudomallei* is gradually expanding to higher latitude regions, which will increase opportunities for the spread of melioidosis. For newly identified high-risk areas, measures such as improving biosafety levels, implementing emergency response, and sharing information can be taken. Especially in the prevention of cross-border animal diseases, the importation of melioidosis can be reduced by strengthening early warning and system construction, improving the animal epidemic monitoring system in high-risk border areas, etc. Comprehensive measures can be taken to effectively control the spread of melioidosis in animals in non-epidemic areas and reduce the impact of the outbreak on public health and animal health.

This study had some limitations. The distribution and modeling results may be influenced by other internal factors, such as distribution distances and dispersal rates of species and their time of generation, and external factors, such as human activities. Furthermore, in ENMs, we only analyzed selected environmental variables, such as temperature and precipitation, whereas soil moisture and hosts were not analyzed. Future investigations will need to address these gaps in the research.

## 5. Conclusions

This study used the MaxEnt model to predict global animal melioidosis risk regions under current and future climate scenarios and discovered that the environmental density of *B*. *pseudomallei*, temperature, and precipitation were important factors influencing the global animal melioidosis risk regions. Under future climate scenarios, the global animal melioidosis risk regions will expand to varying degrees, with a trend toward higher latitude regions. Under current climate conditions, the low-risk regions for melioidosis in most countries will continue to expand in the future. Melioidosis transmission will expand dramatically as global exchanges become more frequent. This work contributes to increasing global awareness of melioidosis prevention, assisting governments in strategically monitoring and controlling melioidosis, and reducing losses.

## Figures and Tables

**Table 1:** Description of the bioclimatic variables included in the maximum entropy model.

Variable	Description	Data Processing	Included
bio 1	Annual mean temperature		N
bio 2	Mean diurnal range		N
bio 3	Isothermality (bio2/bio7)		N
bio 4	Temperature seasonality		N
bio 5	Maximum temperature of the warmest month		N
bio 6	Minimum temperature of the coldest month		N
bio 7	Temperature annual range (bio 5–bio 6)		N
bio 8	Mean temperature of the wettest quarter		N
bio 9	Mean temperature of the driest quarter		N
bio 10	Mean temperature of the warmest quarter		Y
bio 11	Mean temperature of the coldest quarter		Y
bio 12	Annual precipitation		N
bio 13	Precipitation of the wettest month		N
bio 14	Precipitation of the driest month		N
bio 15	Precipitation seasonality		Y
bio 16	Precipitation of the wettest quarter		N
bio 17	Precipitation of the driest quarter		Y
bio 18	Precipitation of the warmest quarter		Y
bio 19	Precipitation of the coldest quarter		N
ED	Density of *Burkholderia pseudomallei* in the environment (soil, water, and air)	Kernel density analysis	Y

**Table 2:** Changes in the areas of risk regions under current and future climate scenarios (10^3^ km^2^).

	2050s			2070s			2090s		
SSPs	Expansion	Stable	Contraction	Expansion	Stable	Contraction	Expansion	Stable	Contraction
SSP 126	18,316.1	28,105.3	5.7	17,901.0	28,099.0	12.0	18,886.7	28,033.3	77.7
SSP 245	23,237.7	28,107.4	3.6	19,265.0	28,105.3	5.6	18,715.6	28,077.0	34.0
SSP 585	19,607.1	28,106.2	4.8	21,805.9	21,808.3	2.7	19,855.5	28,105.3	5.7

## Data Availability

Within the article, we present the data which support our findings.
